# A look into retinal organoids: methods, analytical techniques, and applications

**DOI:** 10.1007/s00018-021-03917-4

**Published:** 2021-08-22

**Authors:** Tess A. V. Afanasyeva, Julio C. Corral-Serrano, Alejandro Garanto, Ronald Roepman, Michael E. Cheetham, Rob W. J. Collin

**Affiliations:** 1 Department of Human Genetics and Donders Institute for Brain, Cognition and Behaviour, Radboud University Medical Center, Geert Grooteplein 10, 6525 GA Nijmegen, The Netherlands; 2 UCL Institute of Ophthalmology, 11-43 Bath Street, London, EC1V 9EL UK; 3 Department of Pediatrics, Amalia Children’s Hospital and Radboud Institute for Molecular Life Sciences, Radboud University Medical Center, Nijmegen, The Netherlands; 4 Department of Human Genetics and Radboud Institute for Molecular Life Sciences, Radboud University Medical Center, Nijmegen, The Netherlands

**Keywords:** Retina, Organoid, Inherited, Omics, Degeneration, Therapy

## Abstract

Inherited retinal diseases (IRDs) cause progressive loss of light-sensitive photoreceptors in the eye and can lead to blindness. Gene-based therapies for IRDs have shown remarkable progress in the past decade, but the vast majority of forms remain untreatable. In the era of personalised medicine, induced pluripotent stem cells (iPSCs) emerge as a valuable system for cell replacement and to model IRD because they retain the specific patient genome and can differentiate into any adult cell type. Three-dimensional (3D) iPSCs-derived retina-like tissue called retinal organoid contains all major retina-specific cell types: amacrine, bipolar, horizontal, retinal ganglion cells, Müller glia, as well as rod and cone photoreceptors. Here, we describe the main applications of retinal organoids and provide a comprehensive overview of the state-of-art analysis methods that apply to this model system. Finally, we will discuss the outlook for improvements that would bring the cellular model a step closer to become an established system in research and treatment development of IRDs.

## Introduction

Inherited retinal diseases (IRDs) comprise a genetically and clinically heterogeneous subgroup of vision disorders with over 270 different causative genes identified so far [1]. Many IRDs are characterised by progressive loss of photoreceptors or the underlying retinal pigment epithelium (RPE) that can lead to severe visual impairment. While significant progress has been made to date, the majority of these blinding diseases remains untreatable.

With the notable example of the FDA approval of a gene augmentation therapy for *RPE65-*associated IRD, recent success in the field of gene augmentation therapies is largely driven by preclinical studies performed in animal models [2–4]. Despite their advantages, such as low cost and ease of genetic manipulation, each animal model also has disadvantages; for example, differences in retinal anatomy, photoreceptor types, or genomic conservation compared to a human, alongside ethical restrictions [5].

In vitro models are becoming an increasingly popular addition to animal models [6]. For example, induced pluripotent stem cells (iPSC) derived from somatic cells of an individual can be genetically reprogrammed to become pluripotent, meaning that they can differentiate into any other adult cell type [7]. Differentiated cells that are generated from these iPSCs contain the unique genome of the individual and, therefore, have the exact genetic and cellular context to study disease mechanisms of hereditary disorders. In the case of photoreceptor-based IRDs, photoreceptors in iPSC-derived retinal organoids will recapitulate many of the pathophysiological processes, both at the cellular and molecular levels that underlie the disease in the respective IRD patients.

The retina is a light-sensitive neural tissue situated at the back of the eye that displays a laminated appearance upon transverse sectioning and light microscopy. At the most posterior part resides the layer of RPE. The basal surfaces of the RPE cells face the choroidal blood vessels, whereas their apical processes interdigitate with photoreceptors. The specialised light-sensing photoreceptor outer segments (OS) form the next layer and are connected to their adjacent biosynthetic inner segments by the connecting cilium. The outer limiting membrane (OLM), a barrier to movement in the extracellular space formed by the photoreceptor and Müller cell end-feet, separates the inner segments from a layer containing the nuclei of rod and cone photoreceptors, called the outer nuclear layer (ONL), whereas photoreceptor axons and processes meet with horizontal and bipolar cells in the outer plexiform layer (OPL). More anterior, the inner nuclear layer (INL) harbours nuclei of the bipolar, amacrine, horizontal, and Müller cells, while the inner plexiform layer (IPL) contains the processes and synapses of bipolar and amacrine with retinal ganglion cells (RGC). The nuclei of RGCs form the RGC layer while their axons form the optic nerve fibre layer. The most anterior layer is populated by astrocytes that form an inner limiting membrane (ILM), separating the retina from the vitreous [8]. During the early embryonic development of the foetal eye, the retina is formed from neuro-ectoderm, the outermost embryonic germ layer. The ectoderm forms two optic vesicles, the distal portion of which folds inward to form the optic cups. The outer and inner walls of an optic cup generate RPE and retina, respectively [9].

**Fig. 1:**
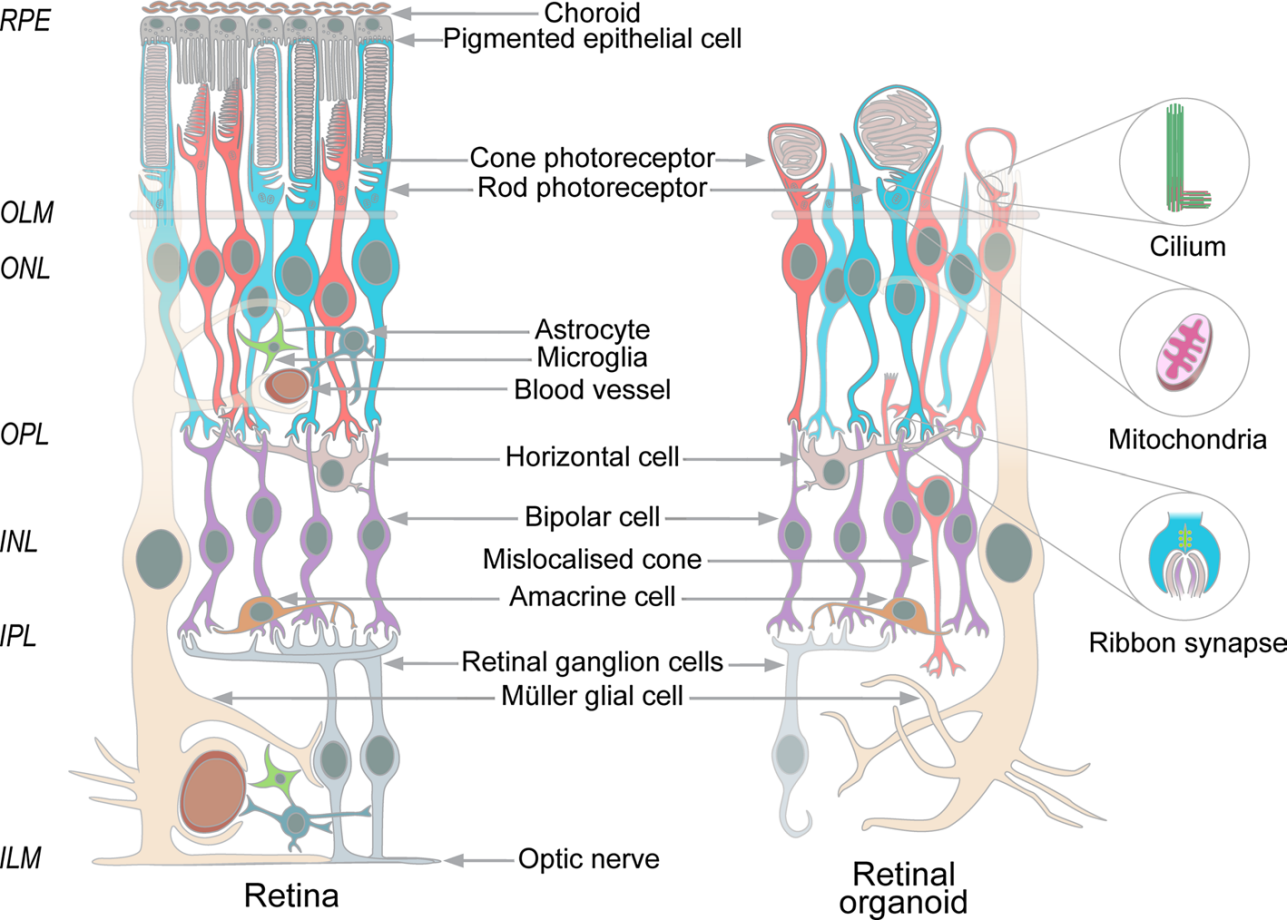
The cellular organization of the retinal organoids mimics the organization of the retina and contains most of the retinal neuronal cell types, such as most posterior RPE (grey), which faces choroidal blood vessels (brown) at the basal and cones (red) and rods (blue) at the apical sides. The outer limiting membrane (OLM) (tan) is formed by the Müller cell end-feet (cream) and photoreceptors. The photoreceptor nuclei constitute a layer called the outer nuclear layer (ONL), whereas their axons and processes meet with horizontal (pink) and bipolar cells (purple) in the outer plexiform layer (OPL). More anterior, the inner nuclear layer (INL) harbours nuclei of the bipolar (purple), amacrine (orange), horizontal cells (pink), and Müller glia (cream), while the inner plexiform layer contains the processes and synapses of bipolar cells (purple), amacrine cells (orange), and RGC that are reduced in number by the stage of photoreceptor maturation (grey). In retinal organoids, subcellular structures specific to the retina were observed such as outer segments (OS), inner segments (IS), and connecting cilia with basal bodies, mitochondria, and ribbon synapses (right panels). In contrast to the retina, the retinal organoids can contain cone photoreceptors in the INL and do not contain choroid, blood vessels, astrocytes (dark blue), microglia (green), and the defined RPE layer

Retinal organoid differentiation resembles the formation of the optic vesicle. After a prolonged culture using state-of-the-art protocols, retinal organoids can contain most of the retinal neuronal cell types such as rods and cones, ganglion, bipolar, horizontal, amacrine, and Müller cells, as well as, mimicking the lamination of the retina with most of the photoreceptor nuclei placed in the ONL, with infrequent misplaced cones appearing in the INL (Fig. 1) [10–17].

**Fig. 2:**
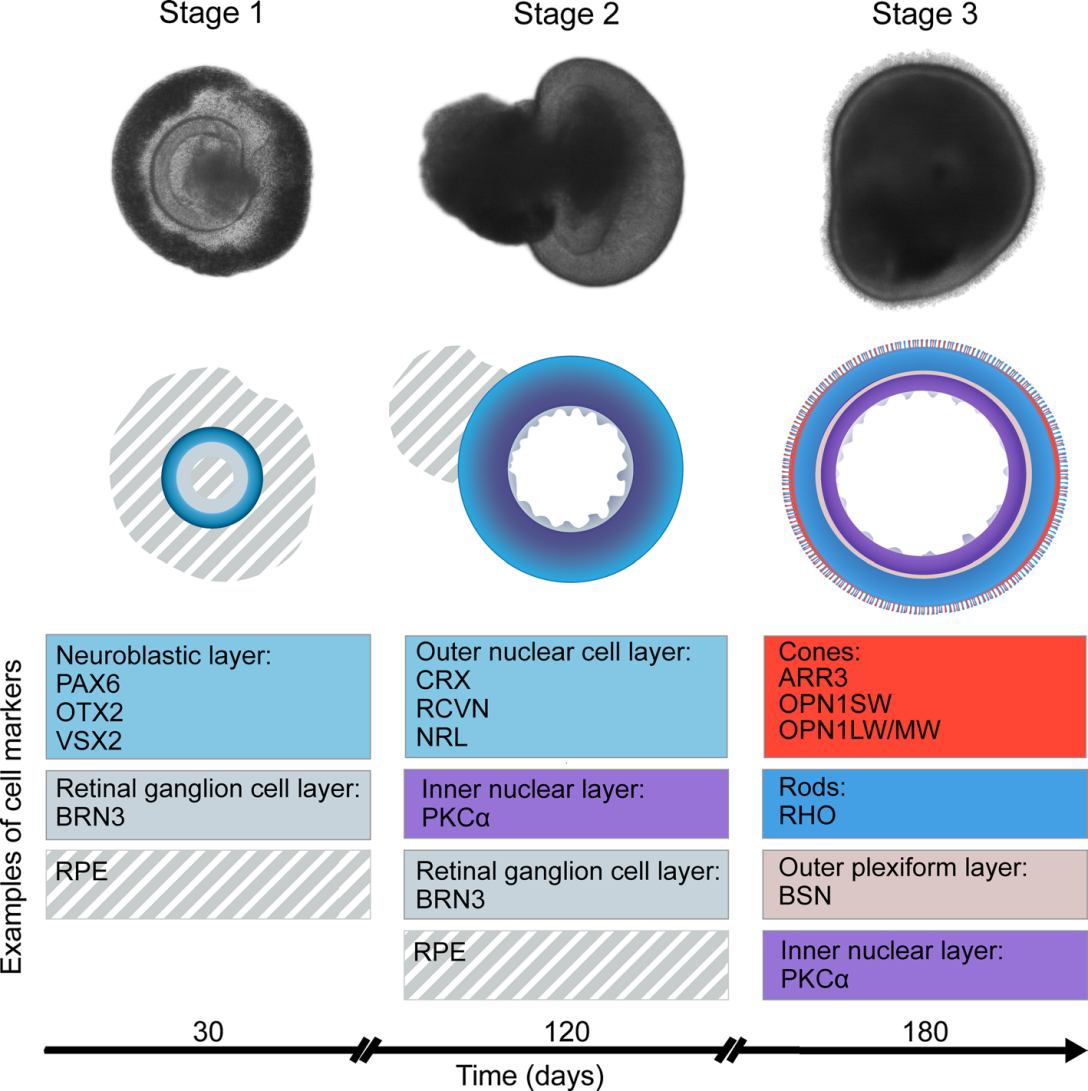
At different maturation stages the retinal organoid contains a progressively complex population of cells. Upper row shows bright field microscopy of retinal organoids at different stages of development. Middle row schematics showing the cellular composition. Lower row shows commonly used markers to identify the different cell types. Colour coding, neuroblastic (pale blue), RGC (grey), inner nuclear layer (bipolar cells, purple), cones (red), rods (blue)

Three differentiation stages of a retinal organoid can be described (Fig. 2). At stage 1, around differentiation day (D) 30 to 50, organoids develop a clear phase-bright outer neuroepithelial rim that contains neural retina progenitors and the inner part of the organoid harbours RGCs. RGCs are the first retinal cells to differentiate at around D50, but their numbers decrease from D90 onwards, seemingly due to the lack of connection to brain targets [11, 17]. A recent paper showed that in assembloid culture, where retinal and brain organoids were fused at D50, RGCs exhibited axonal outgrowth and pathfinding into the cortical neurons of brain organoids, alongside increased proliferation and decreased cell death at D150 [18]. At stage 2, around D80-D120, organoids develop a phase-dark core with a reduced bright rim and early progenitors of cones and rods begin to appear. At stage 3, around D120-180, the outer rim is more visible with hair-like, or brush-border-like structures that correspond to the photoreceptor inner and outer segments [10]. The differentiating retinal organoid expresses a specific set of biomarkers at each maturation stage; for example, photoreceptors express field transcription factor PAX6, and retinal progenitor cell factor VSX2 early in differentiation, followed by a photoreceptor precursor-specific transcription factor CRX, early rod-specific marker NRL, and the mature cone and rod markers recoverin, and L/M/S-opsins and rhodopsin, respectively. Photoreceptor cilium and OS formation during retinal development can be tracked by staining of the PCARE protein from D120-180 [19]. A dark patch of RPE cells is frequently observed as part of the developing neural-retinal vesicle, but not as a monolayer overlying the neural retina. The retinal organoid cell populations are considered to be fully developed between D210 and D260, and decrease in complexity from there on [10, 11].

### Methods of retinal organoid differentiation

Important advances have been made in the development of protocols that are used to differentiate stem cells into retinal cells, improving the formation of well-defined retinal cell types and structures, as well as the efficiency and reproducibility (Table 1).

**Table 1:** Breakthrough methods for differentiation of germline stem cells into retinal organoids

Initial cell type	NRVs formation method	Neural induction media	Retinal differentiation media	Photoreceptor markers	Outer segments	Comment	References
hESCs	EB formation and transferred to adherent culture	D1–D3: DMEM:F12, 10% KSR, B27, 1 ng/mL noggin, 1 ng/mL Dkk1, 5 ng/mL IGF-1	D4–D91: DMEM:F12, B27, N2, 1 ng/mL noggin, 1 ng/mL Dkk1, 5 ng/mL bFGF	RHO, OPN1SW, NRL	Not present	First 2D method to derive retinal cells from embryonic stem cells	[20]
mESCs, mkESCs, *hESCs	EB formation and transferred to adherent culture	D1–D2: DMEM/F12 0.1 mM NEAA, 0.1 mM β-met, 20% KSR, 2 mM l-glutamine D3–D6: G-MEM, 0.1 mM NEAA, 0.1 mM β-met, 20% KSR, 1 mM pyruvate D7–D14: 15% KSR D0–D20: Dkk-1 (100 ng/ml), Lefty-A (500 ng/ml)	D14–D90/D120: GMEM, NEAA, 0.1 mM β-met, 10% KSR, 1 mM pyruvate D90/D120–D200: GMEM, 5% KSR, NEAA, 1 mM pyruvate, 0.1 mM β-met, 1 μM RA, 100 μM taurine, N2	RHO, RCVRN, OPN1LW, OPN1MW, OPN1SW	Not characterised	Inhibition of Notch with DAPT, addition of RA and taurine to increase photoreceptor production	[21]
mESCs	EB formation	D0–D7: GMEM + 1.5% KSR, NEAA, 1 mM pyruvate, 1 mM β-met	D7–D10: DMEM/F12 + N2 D10–D35: DMEM F12 + N2 + 10% FBS + 0.5 μM RA + 1 mM taurine D16: + DAPT	RCVRN, RHO	Not characterised	First 3D method using mouse cells	[23]
hESCs	EB formation	D0–D12: GMEM, 20 uM Y-27632 20% KSR, NEAA, 1 mM pyruvate, 0.1 mM β-met, 3 μM IWR1e D12–D18: + 10%FBS D15–D18: + 3 μM CHIR99021, 100 nM SAG	D18–D121: DMEM/F12-glutamax (3:1), N2, 10% FBS, 0.5 μM RA D121–126: w/o RA	NRL, RCVRN, RHO, OPN1SW	Apical protrusions of mono-ciliated, mitochondria-rich cell bodies, reminiscent of inner segments (ellipsoids), which carry connecting cilia OS formation	Improvement of the 3D method using human cells	[24]
hIPSCs	EB formation	D1: mTesR/NIM (DMEM/F12 1:1, N2, NEAA, 2 μg/mL heparin) 3:1 D2: mTesR1/NIM 1:1 D3–D15: 100% NIM	D16–D41: DMEM/F12 3:1, B27, NEAA D42–D63: DMEM/F12 3:1, B27, NEAA, 10% FBS, glutamax, 100 μM taurine D63–D91: + 1 μM RA D92–end: + 0.5 μM RA	RHO, OPN1LW, OPN1MW, OPN1SW	Present with connecting cilia and basal bodies. Occasionally showing light response in vitro	First 3D/2D method to describe mature and light-responding photoreceptor cells	[17]
hIPSCs	Adherent culture, NRV excision and grown in suspension	D2–D14: DMEM/F12 1:1, NEAA, N2 D14–D21: + 10 ng/mL FGF2 D21: w/o FGF2 D21–D28/D28–D35: + 10 μM DAPT	D14–D112: DMEM/F12 1:1, NEAA, N2	Photoreceptor positive markers in internal rosettes of the organoids: recoverin, OPN1LW, OPN1MW	Positive for the ciliary marker acetylated tubulin. No OS visible	First 2D/3D method including NRV excision	[25]
hESCs	EB formation	D0–D18: 45% IMDM, 45% F12-Ham, glutamax, 10% KSR, 10 μM 1-thioglycerol, 1% chemically defined lipid concentrate D0–D6: + 20 μM Y27632 D6–D16: + 1.5 nM BMP4	D18–D150: DMEM-F12-glutamax, 1% N2, 10% FBS, 0.5 µM RA, 0.1 mM taurine D18–D24: + 3 μM CHIR99021, 5 μM SU5402 (for RPE induction)	NRL, RCVRN positive photoreceptors	Not characterised	Addition of BMP4 increases the formation of neuroretinal epithelium	[30]
hESCs	EB formation	D0–D13/D17: DMEM/F12 + glutamax:neurobasal medium 1:1, 0.5X B27, 0.5 XN2, 0.1 mM β-met, 2 mM glutamax	D13/D17–D27/31: DMEM/F12 (3:1), B27 (w/o vit A), NEAA D27/D31–D187: DMEM/F12 (3:1), B27, NEAA, 10% FBS, 100 μM taurine, 2 mM glutamax	RHO, OPN1LW, OPN1MW, OPN1SW	Present with connecting cilia, basal bodies and membrane disks	Spontaneous formation of neuroretinas	[27]
hESCs	Adherent culture, NRV excision and grown in suspension	D3–D28/D49: DMEM/F12 1:1, N2, NEAA, glutamax	D28/D49–D63: DMEM/F12 3:1, B27, 10% FBS, 100 μM taurine, glutamax D63–D84: + 1 μM RA D84–end: + 0.5 μM RA, + N2	RHO, OPN1LW, OPN1MW, ARR3, PRPH2, RIBEYE, SYNTAXIN3	Present with disorganised disk membranes, connecting cilia and basal bodies	Improvement of the 2D/3D combination model	[26]
hESCs, hIPSCs	EB formation	D1–D6: DMEM/F12 (1:1), N2, NEAA, glutamax, 2 μg/mL heparin D6: + 1.5 nM BMP4	D16–D25/D30: DMEM/F12 3:1, B27, NEAA, glutamax D25/D30–D100: + 5% FBS, 100 uM taurine, 1 μM RA, 1:1000 lipid suplement D100: w/o RA	RHO, RCVRN, OPN1LW, OPN1MW, OPN1SW, NR2E3, NRL, ARL13B, ARR3	Present with partially stacked disks, connecting cilia, basal bodies	Development of a practical staging system of the retinal organoids. Demonstration that BMP4 increases NRV formation. Adaptation from [17] and [30]	[10]
hESCs, hIPSCs	EB formation	D1: E8/NIM 3:1, N2, 2 μg/mL heparin, NEAA D2: E8/NIM 1:1, N2, 2 μg/mL heparin, NEAA D3–D16: 100% NIM	D16–D42: DMEM/F12 3:1, B27, NEAA D42–D63: + 10% FBS, 100 mM taurine, glutamax D63–D92: + 1 μM 9-cis retinal D92-end: + 0.5 μM 9-cis retinal, N2	RHO, OPN1LW, OPN1MW, OPN1SW	Present with connecting cilia and basal bodies	Accelerated photoreceptor differentiation by 9-cis retinal. Adaptation from [17]	[34]
hIPSCs	EB formation	D1–D16: DMEM/F12 (1:1), glutamax, 24 nM sodium selenite, 16 nM progesterone, 80 µg/ml human holotransferrin, 20 µg/ml human recombinant insulin, 88 µM putrescin, NEAA	D16–D40: DMEM/F12 (3:1), B27, NEAA D24: 10 µM Y-27632 D40–D70: + 10% FBS, 100 μM taurine D70–D100: + 1 μM RA D100–D190: + 0.5 μM RA D190–End: w/o RA	RHO, PNA lectin, ROM1	Present with connecting cilia and basal bodies	Generation of retina-on-chip model. Adaptation from [17]	[172]
hESCs	EB formed and maintained in suspension	D1–D18: VNIM (DMEM/F12, 20% KOSR, L-Glutamine, NEAA, B27, IGF-1 (5 ng/mL) D18–D37: + 0.5 uM RA, IGF-1 (5 ng/mL), T3 (40 ng/mL), Taurine (0.1 mM) D1–D2: + 10 µM Y27632	D37-D120: DMEM/F12, L-Glutamine, NEAA, B27, N2, IGF-1 (10 ng/mL)	RCVRN, RIBEYE, RHO, OPN1LW, OPN1MW, OPN1SW	Not characterised	Demonstration that IGF-1 increases the formation of laminated NRVs. Adaptation from [185] following [31]	[32]

The table includes details of the culturing media and reagents used in each protocol for better comparison. Briefly, the majority of protocols use DMEM/F12 as medium base, commonly using the differentiating factors serum, RA, taurine, B27 and/or N2. Non-essential amino acids (NEAA) and glutamax supplements are often included. Small molecules such as IGF1, BMP4, heparin, CHIR99021 or DAPT, may be added at specific time points to regulate differentiation signalling pathways. *RA* retinoic acid, *β-met* β-mercaptoethanol, *NIM* neural induction medium. *Differentiation protocol described for this initial cell type

One of the earliest successful differentiation methods used adherent (2D) cultures to direct embryonic stem cells into an anterior neural fate. Adding Wnt/BMP signalling inhibitors alongside IGF-1 to the media, induced the formation of photoreceptor-marker positive cells, but these were not the main cell types in the adherent culture [20]. Inhibition of Notch signalling with DAPT treatment significantly increased the proportion of photoreceptor and RPE cells, and the addition of the rod-genesis factors retinoic acid (RA) and taurine boosted the number of photoreceptor-marker positive cells [21]. Neural induction media with heparin and chemically defined N2 supplement nudged iPSC to aggregate into embryoid bodies, which then adhered to the surface of the coated culture dish and differentiated towards neural retina [22]. However, the number of photoreceptors obtained under these conditions was low, these photoreceptors were mainly precursor cells, and were distributed in a monolayer of mixed cultures.

A key step to obtain stratified neural retinas was the transition to non-adherent (3D) protocols. Mouse embryonic stem cell (ESC) aggregates cultured in suspension under low-growth factor conditions together with Matrigel matrix improved the formation of optic cups mimicking the embryonic optic cup with apical-basal polarities [23]. The addition of foetal bovine serum (FBS) and the hedgehog agonist SAG augmented retinal differentiation for human stem cells with laminated retinas, expressing markers of all retinal cell types: ganglion, amacrine, bipolar, horizontal, Müller, and photoreceptor cells. In human ESC-derived retinal organoids, electron microscopy (EM) analysis of the photoreceptor cell layer showed mitochondria and rudimentary connecting cilia with basal bodies, only lacking obvious OS [24].

A combination of 3D and 2D protocols that did not require the addition of small molecules differentiated iPSCs to mature and light-responsive photoreceptor cells with rudimentary OS. This was achieved by reducing the RA concentration between D50 and D70, and prolonging the culturing times [17]. Alternatively, a 2D to 3D approach enabled the bypass of embryoid body formation, generating neuroretinal structures in the adherent culture that were excised and further cultured in suspension [25]. These floating neuroretinas formed neural rosettes containing photoreceptors, but without the characteristic lamination of other 3D cultures. Incorporation of the differentiating retinal factors—serum, RA, taurine, and the supplements N2 and B27—permitted generation of photoreceptors with rudimentary OS visible at the edges of the retinal organoid [26]. Interestingly, a different procedure to the 2D and 3D models generated neuroretinas with mature photoreceptors following spontaneous attachment and spreading of epithelial structures, called cysts [27].

Most of these protocols share common media components, but a switch in the timing and addition of certain molecules helped to improve the yield of neuroretinal vesicles obtained. Bone morphogenetic proteins play a role in establishing dorsal/ventral patterning of the retina [28], and specifically, BMP4 is needed for retina specification in mice [29]. The addition of timed BMP4 treatment was shown to increase the self-formation of neuroretinal epithelia [10, 30]. The factor IGF-1 also facilitated the formation of 3D-laminated retinal organoids when added to the media during the first 3 months of differentiation [31, 32]. Nevertheless, this response to BMP4 and IGF-1 activation is iPSC line- and differentiation method-dependent [33]. Addition of 9-cis retinal, instead of the widely used all-trans RA, accelerated rod photoreceptor differentiation in organoid cultures, with higher rhodopsin expression and more mature mitochondrial morphology evident by D120 [34]. For cone specification, thyroid hormone signalling regulation helped to control the fate of cone subtypes in retinal organoids [12]. RGCs usually appear at D40 to D50 after the start of differentiation. Accelerated ganglion cell development within D28 of differentiation was achieved by encapsulating EBs in a 3D Matrigel drop instead of growing in suspension [35].

### Current applications for retinal organoids

Retinal organoid technology offers the possibility to obtain retinal tissue for a wide range of applications and research questions. To illustrate, here we highlight the potential of retinal organoids for therapeutic transplantation and as a model to assess therapeutic strategies.

### Retinal organoids as a source for transplantation

Photoreceptors are often the first cell type lost in many retinal diseases. When that occurs and the adjacent retinal layers remain intact, transplantation of healthy photoreceptors could be a potential treatment option. Photoreceptors derived from human stem cells are an exceptional and unlimited source of human cells for transplantation. Transplanted photoreceptors were first thought to integrate into the ONL of degenerating retina and improve vision in mouse models [36, 37]. However, recent studies have demonstrated that transplanted photoreceptors do not integrate; instead, they remain in the subretinal space and exchange cytoplasmic material with the host cells [38–41]. In light of this discovery, earlier publications should be interpreted with caution and new carefully designed studies are required to clarify if integration or material transfer are mediating the observed effects.

Transplanted embryonic stem cell-derived photoreceptors from 2D cultures into adult *Crx*^*−/−*^ mice retinas were able to produce ERG responses [42]; however, the photoreceptors used in this study did not mature to form OS. The advent of 3D protocols to generate retinal organoids [17, 23, 24] offered the opportunity to obtain a better yield and reproducibility of photoreceptors. For example, transplantation of mouse ESCs-derived photoreceptors by subretinal injection into *Gnat*^*−/−*^ mice displayed features of mature photoreceptors, such as inner and outer segments. When transplanted to *Rho*^*−/−*^ and *Prph2*^*−/−*^ mutants, the donor cells showed rhodopsin and PRPH2 positivity that were lacking in the endogenous retinas, although they did not contain OS [43]. Transplantation of cones purified from retinal organoids into adult *Nrl*^*−/−*^ mice, a model where rod cells take a different fate and become S-cones [44] was reported to be successful [26]. Donor L/M cones were well oriented in the ONL and expressed markers of the mature cell type. In adult an LCA4 model, *Aipl1*^*−/−*^ mice that lack photoreceptors transplanted L/M cones expressed the presynaptic protein ribeye and photopigments. However, the functionality of these cells was not assessed [45].

The source and developmental stage of transplanted cells, as well as their enrichment and delivery methods, are key to successful transplantation. The body of photoreceptor precursor cell isolation and transplantation work suggests that fully mature photoreceptors may be more fragile and unfit for transplantation than retinal progenitor cells. Fluorescence-activated cell sorting (FACS) of the progenitor cell marker C-kit, a type III receptor tyrosine kinase, and the pluripotency marker SSEA4, can be used to isolate retinal progenitor cells without tumorigenic components [46, 47]. C-Kit^+^/SSEA4^−^ cells possess the characteristics of retinal progenitors and in *rd1* mice, organoid-derived C-kit^+^/SSEA4^−^-transplanted cells protected the ONL and improved visual function. The photoreceptor-specific cell surface markers CD24 and CD73 can also be used to isolate photoreceptors [48, 49]. Label-free techniques of photoreceptor selection that comply with current good manufacturing practices (cGMP) are preferred for clinical translation. Magnetic activated cell sorting (MACS) based on CD73 expression allows for enrichment of rod photoreceptor precursors [50]. Photoreceptors from retinal organoids can also be isolated using a panel of biomarkers [51]. Microfluidic enrichment of photoreceptor precursor cells is a more recent approach that allows to separate RPE cells from the rest of retinal cells based on their mechanical and physical properties. The device was also able to separate some rods from cones, although further improvements will be needed to filter bipolar and ganglion cells [52]. Depending on the disease, the stage of the disease, and the cell type to be transplanted, different delivery methods need to be considered. Trans-scleral and transvitreal injection are effective methods to deliver retinal cells into the eye, with the transvitreal approaches preferred in the clinic [53]. Integration and survival of photoreceptor cells in suspension is limited compared to the more successful transplant of RPE cells [41, 54, 55].

Transplantation of retinal sheets is an alternative to single-cell suspension transplants. Transplanted mouse iPSCs-derived retinal sheets can survive in the subretinal space of *rd1* mice and form synaptic connections with the host retina [56]. The hESC-derived retina can mature and form an ONL with photoreceptor inner and outer segments after transplantation in monkeys and nude rats [57]. The same group demonstrated that the transplanted cells were able to respond to light [58]. Transplanted sheets formed neural rosettes and improved visual function in the host retina of immunodeficient Rho^*S334ter−3*^ rats, a model of severe retinal degeneration [59]. hESC-derived D60-D70 full retinal organoids implanted into the subretinal space of immunosuppressed wild-type cats integrated and formed cytoplasmic and synaptic interactions with the host tissue [60]. Transplantation of iPSCs-derived retinas formed rosettes bearing photoreceptors with OS, bipolar, and amacrine cells, but no RGCs. Grafted cells formed synapses with the host bipolar cells and generated light-responses. These cells survived after 5 months in rats, and over 2 years in monkeys, demonstrating the long-term potential of this therapeutic approach [61].

Other physical factors may play an important role in transplantation. The OLM is made of cadherin–catenin complexes that mediate cell–cell adhesion and serves as a barrier for the integration of transplanted photoreceptor precursor cells [62]. Disruption of OLM proteins enhanced integration of transplanted photoreceptors [63]. Gliosis caused by reactive Müller cells might be considered as well in the context of transplantation and OLM as a barrier [64].

Despite these advances and the availability of well-characterised GMP-compliant retinal cells via stem cell differentiation, there are still many challenges to be overcome to achieve the level of successful transplantation that is needed to restore high acuity vision.

### Retinal organoids to assess therapeutic strategies

Therapies based on adeno-associated virus (AAV) vectors are gaining momentum as a potential treatment for retinal diseases. One major reason is the accessibility of the eye, which makes it suitable for intravitreal or subretinal injection surgery. AAVs can infect human cells, allowing long-term expression of the transgene after a single dose.

There are several known AAV serotypes [65], with different tropisms for the tissue they can infect. AAV serotype 2 has been widely used in the eye [66]. Human retinal organoids offer an opportunity to investigate viral tropism and transduction in tissue that resembles the human retina. The first evaluation of different AAV serotypes in retinal organoids showed that AAV2/8 and ShH10 (an AAV6 variant) [67] were the most robust to transduce photoreceptors in this system, with higher efficiency of the latter [68]. The promoter rhodopsin kinase was able to drive expression in both rod and cone photoreceptors, while the opsin 2.1 promoter was active in L/M-cones and some S-cones [68]. The AAV serotype AAV2-7m8 in combination with opsin 1.7 promoter was effective in the transduction of L/M-cones [69]. Compared to AAV2, AAV9, and AAV8, AAV2-7m8 demonstrated higher tropism for photoreceptor precursor cells at D44 of differentiation and remained stable for at least 4 weeks [70]. AAV2/5 under the CAG promoter transduced photoreceptors with high efficiency (~ 90%) and was used to rescue RP2 expression in RP2-KO retinal organoids, improving photoreceptor survival [71]. On the retina-on-chip model, the AAV2-7m8 variant was also more efficient at transducing mature photoreceptors, Müller, and ganglion cells, compared to first-generation AAV variants [72]. The newly generated recombinant AAV vectors AAV2.NN and AAV2.GL outperform AAV2 and AAV2-7m8 in mice and are able to effectively transduce human retinal explants [73]. In organoids, the variant AAV2.GL had a comparable transduction efficiency to AAV2-7m8, while AAV2.NN had higher fluorescence levels than all other AAV variants tested [72]. The NN peptide-insert also showed high transduction efficiency of organoids when used in the AAV9 vector [74].

Delivery of AAV by intravitreal injections is a safe method, but it often leads to low transduction efficiencies of photoreceptors. A novel injection system using peripapillary intravitreal injection promises to be a safe and efficient alternative to standard intravitreal injections [75]. This, together with recent positive results on retinal-transduction efficiency of newly designed second-generation AAVs, holds hope for the future of AAV-based retinal therapies when combined with intravitreal delivery.

Another type of therapeutic strategies are RNA-based therapies, such as antisense oligonucleotides (AONs), which are becoming popular to treat IRD [76, 77]. AONs are relatively small nucleic acid molecules that target the pre-mRNA or mRNA to modify the splicing process, alter translation or degrade a transcript. Currently, four clinical trials using these molecules are ongoing for *CEP290-* (NCT03913130 and NCT03913143), *USH2A-* (NCT03780257), and *RHO-* (NCT04123626) associated IRD. Retinal organoids have been instrumental to evaluate the efficacy and safety of several AON molecules that modulate splicing to either correct defects introduced by deep-intronic variants in *CEP290* [78, 79] and *ABCA4* [80] or to create shorter proteins with a residual function in *USH2A* [81]. This implementation of retinal organoids will be further discussed in “Splicing studies” section.

### Analyses of retinal organoids

**Fig. 3:**
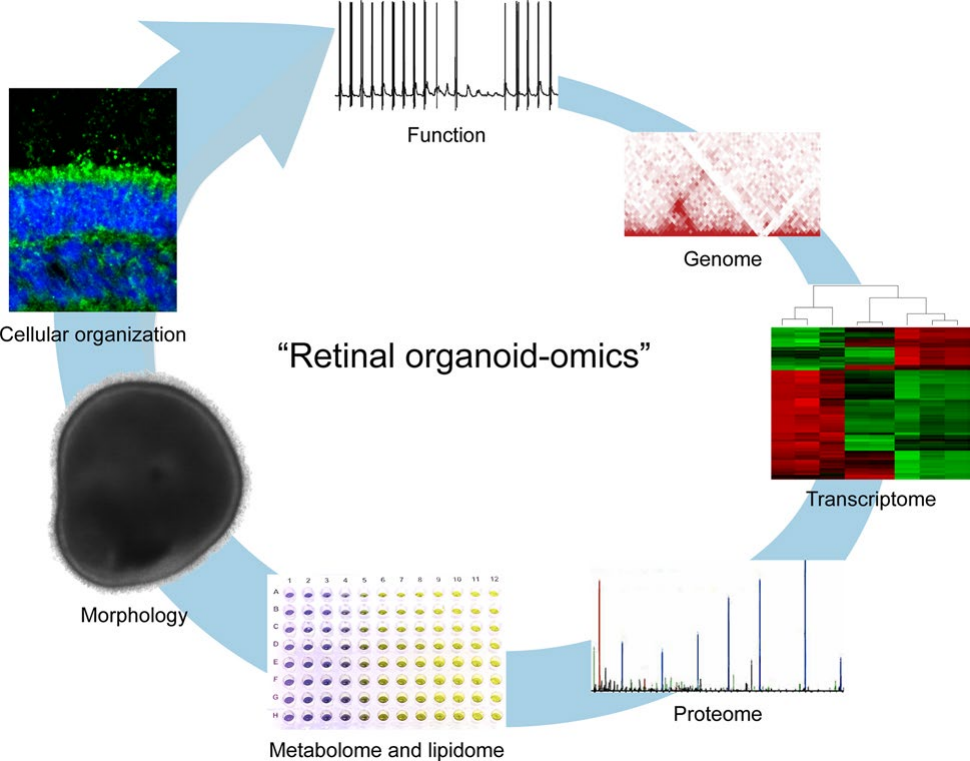
Retinal organoids can be analysed at multiple levels of complexity such as at gene, protein, metabolome, morphology, cellular and subcellular organizations, and finally—function

The advent of new analytical methods that include and integrate multiple next-generation “omics” technologies is on par with the progress of organoid technology (Fig. 3). This facilitates the extraction of useful information from organoids, or a subpopulation of cells, at different levels of complexity and detail.

### Genome analysis

The epigenetic landscape and intragenic/intergenic variants play an important role in regulating gene expression. One such epigenetic feature is a 3D organization of chromatin in the nucleus. The chromatin organization can be examined at a high spatial resolution, using methods such as chromosome conformation capture (3C) assays. In these assays, first the chromatin-deoxyribonucleic acid (DNA) complexes in the nucleus are crosslinked with formaldehyde. Then, the tissue is fragmented, and the DNA is extracted, ligated, and digested with restriction enzymes. Interacting loci are amplified with polymerase chain reaction (PCR) and quantified by sequencing methods [82, 83]. A high-throughput assay called Hi-C allows mapping of chromatin interaction genome-wide in the tissue of interest, in this case, the retina for IRD. For example, the so-called low-input Hi-C analysis of retinal organoids was used to develop the first topologically associated domain (TAD) structure from the human retina and then examine the consequences of an IRD-associated structural variant. This analysis revealed a disrupted chromatin organization with repositioning of domain boundaries, formation of a neo-TAD, and consequent misexpression of *GDPD1* in the RP17 locus of patients with autosomal dominant retinitis pigmentosa (RP) [84]. The low input Hi-C method allows for low amounts of input material and thus mitigates one of the largest hurdles of the retinal organoid system [82], suggesting it could be used to study other IRDs with a genomic reorganisation.

Another promising application of retinal organoids for epigenetic analysis might be chromatin accessibility assay via the transposase-accessible chromatin using sequencing (ATACseq). ATACseq can identify regions of open chromatin and cell-specific transcription factors binding sites via the action of hyperactive Tn5 transposase and high-throughput deoxyribonucleic acid (DNA) sequencing [85]. It was previously implemented in patient-derived RPE cells to reveal decreased chromatin accessibility in age-related macular degeneration (AMD), which is the most common cause of central vision loss in the elderly [86, 87].

The small size and heterogeneity of cell types in retinal organoids pose significant technical challenges for techniques with higher material demand, lower sensitivity, or difficulty to distinguish between the signal coming from different cell types; this is also true for epigenetic analyses. However, for a disease where a primary tissue is difficult to obtain, such as in IRDs, organoids provide a unique opportunity to study the epigenetic landscape of a specific patient genome in the correct cellular context.

### Transcriptome analysis

The retinal organoid system can also be used to quantify the changes in expression levels of retina-specific genes between a patient, or gene-edited line, and control, or following a therapeutic intervention. For this, ribonucleic acid (RNA) is converted into single-strand complementary DNA (cDNA) using a retroviral enzyme, reverse transcriptase with either oligo-dT for priming from the poly-A tails or random hexamers. The cDNA is used as a template for amplification of the gene of interest with PCR [88]. The resulting amplicon is then visualised with an agarose gel electrophoresis in reverse-transcription PCR (RT-PCR), or fluorescence can be incorporated for detection of amplicons during the early exponential phase of the PCR, in so-called quantitative PCR (qPCR) [89]. Pioneering studies used the qPCR technique to show that the temporal profile of neural retinal markers expression of mouse ES-derived organoids follows that of the retina in vivo [23]. qPCR is sensitive even for low abundant genes and requires little starting material; therefore, it remains widely used for gene expression analysis in retinal organoids. To detect targets with a very low abundance, droplet digital PCR (ddPCR) can be employed, which is a water–oil emulsion droplet-based method where a sample is split into droplets, and PCR amplification occurs in each individual droplet. This technique was used to quantify the correction of the aberrant splicing in retinal organoids derived from a Leber congenital amaurosis 10 (LCA10) patient post AON treatment [79].

### Retinal development mapping

A snapshot of all RNA transcripts can be identified with microarrays or RNA-sequencing (RNA-seq). Microarray analysis utilises a collection of specifically designed short DNA oligos, which gives a quantifiable fluorescent signal upon hybridization with a cDNA from retinal organoids. With microarray analysis, the rhodopsin-positive cells of D26 and D34 ESC-derived organoids were compared to P12 early postnatal mouse photoreceptors, revealing that D34 were more mature and closer resembling P12 photoreceptors [43]. These arrays are cost-effective and require a small amount of sample material [90].

The array technique allows for transcriptome-wide gene expression profiling, including the ability to detect alternative splicing events, whereas RNA-seq allows for the identification of a larger number of genes than microarrays and is also applicable to unknown transcripts [91]. Here, the cDNA fragments are used for construction of an RNA-seq library, which then is compared to a reference transcriptome after next-generation sequencing. Bulk RNA-seq profiling demonstrated a gradual and continuous increase of photoreceptor-specific genes in developing retinal organoids, with cone-specific markers expressed before rod-specific ones [92]. Also, cone cells were shown to exist at different stages of maturation within a single organoid [93]. RNA-seq was instrumental to confirm that genes related to visual perception, synaptogenesis, and phototransduction increase over time in developing retinal organoids, and their expression is similar to late foetal and adult retinal transcriptomes [34].

The averaged gene expression profiles resulting from the bulk RNA-seq analysis limit its applicability to the mixed cell populations that comprise a retinal organoid. The gene expression profiles of individual cells yielded by single-cell RNA-seq (scRNA-seq), on the other hand, allow expression data to be refined to specific cell types [94]. ScRNA-seq profiling identified photoreceptor populations [95] and also distinguished between rods and cones [96] in mature retinal organoids. scRNA-seq of ten pooled organoids at D220 revealed four major groups of cells: rods, cones, Müller cells, and a mixed population of retinal neurons, including bipolar, retinal ganglion, and amacrine cells [97]. scRNA-seq data from 4 different maturation stages of retinal organoids and bulk RNA-seq of retinal tissue at 16 stages of human retinal development were compared to the mouse transcriptome and revealed species-specific differences of gene expression in specification of cone photoreceptors and horizontal interneurons and gene regulatory networks that pattern the macula [98]. The mature retinal organoid transcriptomes were closer to peripheral than to foveal adult human retina and contain the largest number of diverse cell types at D210-266, and those cell types appear in the following temporal order: ganglion cells, photoreceptor precursors, horizontal cells, amacrine cells, bipolar cells, and Müller cells [11]. Another study used scRNA-seq to show that retinal organoids are similar in cellular composition to the foetal retina at equivalent stages of development; however, differences in levels of gene expression and quality of lamination were observed. Interestingly, lamination was superior in dissected foetal retina that was cultured under the same conditions as retinal organoids for up to 5 months. The study also described a transition cell population that is situated between progenitor and differentiated cell populations [15]. Furthermore, retinal organoid scRNA-seq has been used to investigate the potential for CRISPR-based genome editing in the retina. Downregulation of genes associated with the homology-directed repair (HDR) pathway (that are crucial for accurate genome editing) after cell division was reported in both adult mouse and human retinas as well as retinal organoid datasets, whereas non-homologous end joining (NHEJ) and microhomology-mediated end joining (MMEJ) pathways were still active, suggesting NHEJ or MMEJ are more likely to be successful than HDR [99]. Nevertheless, despite the power of scRNA-seq the method is continually developing and advances are still needed in read depth, length, and improved sensitivity for the in-depth understanding of gene expression at the single-cell level in organoids.

### Isogenic controls

Several studies have compared the expression of a gene of interest in patient-derived organoids versus controls. Organoids derived from a patient with splicing factor-related RP showed differential expression of a number of genes related to ciliary structure and photoreceptor morphology [100]. RNA-seq analysis of patient-derived retinal organoids harbouring mutations in *AIPL1*, underlying severe inherited retinal dystrophy Leber congenital amaurosis type 4 (LCA4), unexpectedly showed no difference in gene expression of *AIPL1* itself or that of other retinal specific genes, but it did of the cortical development gene *NEUROD6* at D25, D60, D88, and D123; furthermore, in the patient-derived organoids, photoreceptors developed normally [101]. In contrast, scRNA-seq of organoids at D100 and D170 bearing a loss of NRL protein confirmed downregulation of rod-specific genes [14].

A gene expression profile of patient-derived retinal organoids can be compared with that of an isogenic control generated by CRISPR/Cas9-mediated correction, thereby reducing inter-individual variation and more precise control of phenotypes associated with a disease-causing mutation. For example, retinal organoids bearing an RP-causative mutation in RPGR showed upregulation of necrosis and inflammation receptors when compared to both isogenic and unrelated controls [102]. Similarly, in organoids with a mutation in X-linked juvenile retinoschisis causative gene *RS1*, the expression of *RS1* alongside retinopathy-associated genes *IQCB1* and *OPA1* was reduced compared to isogenic control organoids [103]. Alternatively, an isogenic pair can be created by CRISPR/Cas9-mediated gene knock-out in a healthy control iPSC line. A CRISPR-generated knock-out of both isoforms of the nuclear hormone receptor thyroid hormone receptor β (Thrβ2 and Thrβ1), but not the Thrβ2 alone, as was previously thought, directed the cone fate specification toward the S-opsin type [12]. The *RP2* gene encodes for a GTPase-activating protein linked to a severe form of X-linked RP. Isogenic disruption of RP2 in retinal organoids shared expression profile similarities to patient-derived organoids, including upregulation of a number of pro-apoptotic genes compared to isogenic controls that correlated with photoreceptor cell loss [71].

### Splicing studies

In the retinal organoid system, RT-PCR and RNA-seq can also enable mapping of developmental switches in splicing and give an insight into the retina-specific alternative splicing events, where several distinct mRNAs are produced from the same gene, leading to different isoforms. Photoreceptors have a high level of alternative splicing with many unique or enriched isoforms. The alternative splicing programme in photoreceptors is independent of *Crx* and it is driven, amongst others, by the protein Musashi-1, which promotes the inclusion of photoreceptor-specific exons [104, 105]. Alternative splicing can also be associated with pathogenic events. For example, the levels of aberrant splicing and pseudoexon inclusion associated with a deep-intronic variant in *CEP290*, which causes LCA10, were higher in patient-derived retinal organoids compared to RPE or other cell types [78]. Retinal organoids were used to show that AONs could correct the aberrant splicing in LCA10 [78, 79] and this approach shows promise in a clinical trial [106].

Mutations can also occur in retinal specific exons; for example, RT-PCR of control retinal organoids showed an increase in expression of the alternatively spliced isoform *REEP6.1*, which harboured a mutation in a family with autosomal recessive RP, correlated with the time course of photoreceptor development [107]. Similarly, expression of a retinal isoform of the ciliopathy and RP-linked gene *DYNC2H1* increased in maturing retinal organoids over time to become the dominant transcript at D200, correlating with the non-syndromic RP in patients with mutations affecting the retinal isoform only [108]. In Stargardt disease, an *ABCA4*-associated progressive disorder of the retina that is initially characterised by a loss of central vision, retinal organoids exhibited enhanced splicing defects, such as pseudoexon insertion caused by deep-intronic variants, compared to transfection of gene constructs in non-retinal cells, and were used to show the therapeutic potential of an AON for splice correction [80]. RNA-seq analysis of *PRPF31* patient organoids revealed changes in alternative splicing of key components involved in the splicing process, as well as part of the microtubule and ciliary networks [100]. CRISPR/Cas9-mediated correction of a *PRPF31* mutation in retinal organoids and RPE rescued these molecular and cellular defects [100].

To conclude, transcriptome analysis via the RNA-seq method has become a pivotal tool in the analysis of retinal organoid development and disease mechanisms. The disadvantage of RNA-seq is the difficulty in reconstructing full-length transcripts from the assembly of reads, particularly when alternative splicing into highly similar isoforms occurs. Long-read sequencing provides amplification-free, single-molecule sequencing of cDNAs and eliminates the need for the library assembly step, and thus can be more sensitive for the alternative splicing events in distinct cell populations of the retinal organoid [109, 110]. Other limitations of RNA sequencing techniques include sensitivity to the number of reads and underrepresentation of the reads from short and low abundant genes. In addition, a high cost along with a variety of protocols and analysis methods might make RNA-seq techniques challenging to implement.

### Proteomic analysis

An increase in RNA transcript numbers does not necessarily lead to an increase in the amount of the encoded protein. RNA or protein stability and post-translational modifications also contribute to protein levels [111, 112]. Western blotting (WB) is an electrophoresis-based protein detection technique that utilises a polyacrylamide gel matrix for size separation of proteins followed by staining of immobilised antigens on immunoblots by using highly specific antibodies [113]. Several studies employed WB to demonstrate a presence of an additional version of a protein, such as TRNT1 [114], decrease of protein expression, such a RS1 [103], and AIPL1 [101] in patient-derived retinal organoids versus control, or rescue of protein expression after treatment [78, 115].

Due to the small size of the laminated retinal organoid, it is challenging to obtain sufficient material for WB analysis. Although one study reports pooling together at least 16 stage-1 (D18-D35) organoids per condition [116], this approach might be even more challenging for later stage organoids due to their small yield, and time- and labour-intensive differentiation protocols to produce sufficient material for robust replicates. A reverse-phase proteomic array (RPPA) that requires nanogram-range amounts of proteins could be implemented to study retinal organoids. In this technique, protein extracts are printed onto nitrocellulose-covered microscope slides and then quantified using antibodies [117].

A more exploratory technique, mass spectrometry (MS) analysis can identify and quantify multiple proteins in complex mixtures by measuring the mass-to-charge ratio of peptides that are labelled with isotopes [118, 119]. For example, MS suggested a role of differential splicing to be more significant in RPE than in organoids [100]. However, the sensitivity of the MS might be lower in retinal organoids due to its cell population heterogeneity.

### Metabolomic analysis

Metabolomics is another -omics approach that allows sensitive and semi-quantitative detection of low molecular weight molecules at a given time. The “metabolome” can rapidly change and reflect the dynamics of a biological system [120]. While metabolomic studies are not very frequent in the area of ophthalmology (recently reviewed in [121] and [122]), they are well-implemented tools for new-born screening and diagnosis of many multisystemic diseases and currently being further developed to find new disease indicators and biomarkers that allow following the progression of a disease or the effect of therapeutic intervention [120]. Using small intestine and pancreas organoids, a study concluded that the organoid system does not recapitulate the full functional metabolomic spectrum of the organ and suggested improvements in the cultivation process [123]. However, the progress in intestinal development has allowed performing metabolic studies in a more robust and reliable manner [124–126]. Unfortunately, this type of study has not yet been fully explored in the retinal organoid field, as shown by the limited literature available.

Imaging techniques, such as hyperspectral imaging (HSpec), can be used to assess the metabolic activity of the organoids. HSpec showed that retinol and RA signal intensity increased following an increase in CRX expression. The metabolic state can also be imaged with fluorescence lifetime imaging microscopy (FLIM) that shows glycolytic activity by measuring free and bound NADH distribution. In D150 organoids, the glycolytic region appeared in the laminated outer edge that corresponds to the emergence of a highly metabolically active photoreceptor layer [127]. Recently, a dynamic full-field optical coherence tomography (D-FFOCT) was developed where backscattering from cell dynamics or metabolic activity resulted in higher resolution compared to conventional OCT. This technique could distinguish between RPE and photoreceptor progenitor populations in early-stage organoids [128]. These techniques show promise for the non-invasive determination of the retinal organoid maturation stage; however, it will be important to fully correlate the analysis results with the large array of cell type markers via widely-established immunohistochemical methods.

Interestingly, for retinal organoids, an easy and renewable source of material is the culture medium. Many factors are released to the medium, and they could provide valuable information about the stage of the organoids and the active functions. Furthermore, the used medium is usually discarded and thus the metabolomic studies throughout the complete retinal organoid differentiation could be established. For example, high-performance liquid chromatography (HPLC) system coupled to MS allowed the analysis of the media content of retinal organoids treated with retinotoxic antibiotic moxifloxacin [129]. Another possibility could be to study the complement system in AMD organoids. In a recent study, the authors performed a quantitative multiplex profiling of all complement components to diagnose disease [130]. Thus, analysing the secretome of AMD organoids using the medium, could provide a correlation with the disease to what has been observed in human biofluids. An alternative targeted method for a specific group of biomarkers could be an enzyme-linked immunosorbent assay (ELISA). This immunoassay can quantitatively measure a marker of interest; however, well-validated and robust antibodies need to be used to ensure reliable measurements [131]. ELISA could be useful to assess the levels of specific components of the complement pathway, as demonstrated recently by a study in plasma of AMD patients [132, 133]. However, the composition of the medium might interfere with the results. For example, if the aim is to study the metabolism of specific amino acid, sugar, protein, or a growth factor if it is already included in the culture medium, it may mask the possible effect. Altogether, exhaustive development might be needed to apply metabolomics as a regular -omics strategy for retinal organoids.

### Lipidomic analysis

Lipidomics is another -omics strategy based on measuring the different types of lipids by combining extraction protocols with detection methods such as liquid chromatography-mass spectroscopy. In the past, lipids in the retina of multiple species have been exhaustively studied [134–141]. Depositions of lipids are found in patients suffering from AMD (drusen) or Stargardt disease (lipofuscin). Furthermore, several IRD-linked genes have been associated with lipid metabolism, such as *ELOVL4* and *CERKL*. In addition, sphingolipidosis, such as Gaucher disease, or complex lipid disorders like Sjögren-Larsson syndrome, also present a retinal phenotype. Thus, lipidomics is a very appealing approach to study the mechanism of disease in cellular models. Retinal organoids can be used to study how changes in lipid metabolism affect retinal health; for example, deoxysphinganine-induced photoreceptor apoptosis in retinal organoids, supporting the potential association of serine and lipid metabolism disruption in macular telangiectasia type 2 [142].

### Morphological analysis

Most studies of retinal organoids rely on immunohistochemistry (IHC) as a gold standard for characterisation of differentiation and identification of protein and cellular changes [143]. This microscopy-based technique visualises protein expression and localization by antibody staining. First, a cross-linking fixative, such as paraformaldehyde, is used to preserve the cellular architecture and to immobilise target antigens. The fixation agent can degrade or mask an antigen or epitope of interest; therefore, the optimal fixation and staining conditions should be experimentally determined for each antigen–antibody combination separately. Subsequently, the organoids are cryopreserved with sucrose or similar substance, then embedded in suitable media such as OCT, frozen, and sectioned to obtain thin slices that are eventually subjected to antibody staining. Perpendicular sectioning and careful positioning of the organoids is required to capture the photoreceptors in the correct orientation. A list of commonly used antibodies is provided in Table 2.

**Table 2:** A wide range of antibodies is available for IHC of retinal organoids

Antibody (antigen)	Usage	Host	Supplier	Catalog number	Dilution	References
ABCA4	IRD-causative gene OS protein	Mouse	Rockland Immunochemicals	200301D05	1/100	[11]
Acetylated tubulin	Cilia, inner segment, stages 2,3	Mouse	Sigma-Aldrich	T7451	1/1000	[150]
AP2α	Neural crest marker, stage 1	Mouse	DSHB	3B5	1/35-1/100	[101, 150]
AP2α	Neural crest marker, stage 1	Mouse	Santa Cruz Biotechnology	sc12726	1/100-1/200	[32]
AQP1	Cilia, inner segment, stages 2,3	Rabbit	Millipore	ab2219	1/500	[182]
ARL13B	Cilia, inner segment, stages 2,3	Rabbit	Protein Tech	11,711	1/200-1/1000	[10, 11, 101, 182]
ARL13B	Cilia, inner segment, stages 2,3	Mouse	Abcam	ab136648	1/1000	[71]
ARR3	Cone photoreceptor, stage 3	Rabbit	LS Bio	LSC368677	1/300	[10]
ARR3	Cone photoreceptor, stage 3	Goat	Novus Biologicals	NBP137003	1/200	[10, 11]
ARR3	Cone photoreceptor, stage 3	Rabbit	Novus Biologicals	NBP241249	1/1000	[101]
ARR3	Cone photoreceptor, stage 3	Goat	Santa Cruz Biotechnology	sc54355	1/50	[173]
Bassoon	Synapse protein, stage 3	Rabbit	Cell Signalling Technology	6897S	1/200	[34]
Bassoon	Synapse protein, stage 3	Mouse	StressGen	PS003	1/100-1/200	[31, 32]
Bassoon	Synapse protein, stage 3	Mouse	Enzo	SAP7F407	1/500–1/800	[11, 71]
BEST1	RPE	Rabbit	Abcam	ab2182	1/250	[160]
BEST1	RPE	Mouse	Novus Biologicals	NB300164	1/1000	[150]
BiP	Endoplasmic reticulum chaperone	Rabbit	Abcam	ab21685	1/1000	[160]
BrdU	Proliferation marker, S phase, retinal progenitor cells, stage 1	Rat	Accurate	OBT0030	1/250	[144]
BRN3A	RGC, stages 1,2	Mouse	Millipore	ab1585	1/200-1/250	[34, 150, 160, 182]
BRN3B	RGC, stages 1,2	Goat	Santa Cruz Biotechnology	sc31989	1/50	[72]
Calbindin D-28K	Horizonal or amacrine cells	Rabbit	Millipore	ab1778	1/200-1/300	[31, 32]
Calbindin D-28K	Horizonal or amacrine cells	Rabbit	SWANT	CB38	1/500	[101]
Calbindin D-28K	Horizonal or amacrine cells	Rabbit	Calbiochem	PC253L	1/500	[34, 182]
CaR	Amacrine cells and RGC, stage 1	Rabbit	Millipore	ab5054BD	1/500	[10]
CaR	Amacrine cells and RGC, stage 1	Rabbit	SWANT	CR7697	1/500	[101]
CD73	Surface antigen, photoreceptors, stage 2	Mouse	BioLegend	AD2/344002	1/100	[50, 150]
CD73-FIT	Surface antigen, photoreceptors, stage 2	Mouse	BioLegend	AD2/344015	1/1000	[50]
CHAT	Starburst amacrine cells, stage 1	Mouse	Millipore	ab144P	1/100-1/300	[10, 11]
CRALBP	Müller glia, stages 2,3	Mouse	Abcam	ab15051	1/250-1/500	[10, 11, 34, 72, 101, 173, 182]
CRALBP	Müller glia, stages 2,3	Mouse	Invitrogen	MA1813	1/300	[71]
CRX	Photoreceptor progenitors, stages 1,2	Rabbit	GeneTex	GTX124188	1/1000	[144]
CRX	Photoreceptor progenitors, stages 1,2	Mouse	Abnova	H00001406M02	1/100-1/5000	[10, 31, 50, 101, 150]
CRX	Photoreceptor progenitors, stage 1,2	Rabbit	Atlas Antibodies	HPA036763	Jan-50	[160]
CTBP2	Ribbon synapse, Stage 3	n/a	BD Biosciences	612,044	1/300	[10]
EZRIN	Pigment epithelial cells	Rabbit	Cell Signalling Technology	3145S	1/200	[173]
EZRIN	Pigment epithelial cells	Mouse	Sigma-Aldrich	MS661	1/250-1/500	[150, 182]
G0α	Rod and cone ON bipolar cells, stage 3	n/a	Millipore	Mab3073	1/700	[10]
GNAT1	Rod photoreceptors, stage 3	n/a	GeneTex	GTX114440	1/500	[72]
GT335	Cilia, inner segment, stages 2,3	Mouse	Adipogen Life Sciences	AG20B0020	1/800	[71]
HuC/D	Amacrine cells and RGC, stages 2,3	Mouse	Molecular Probes	a21271	1/200	[31, 32] [144]
Islet1/2	RGC, stages 1,2	Rabbit	Santa Cruz Biotechnology	sc30200	1/200	[31]
KDEL	Endoplasmic reticulum marker	Mouse	Abcam	ab176333	1/500	[160]
Ki67	Proliferation marker, G1, S, G2, and M phase, retinal progenitor cells, stage 1	Mouse	BD Biosciences	550,609	1/200-1/500	[50], [144]
Ki67	Proliferation marker, G1, S, G2, and M phase, retinal progenitor cells, stage 1	Rabbit	Abcam	ab15580	1/100-1/500	[10, 31, 32, 50]
LAMP2	Lysosomal marker	Mouse	Santa Cruz Biotechnology	sc18822	1/50	[173]
LHX2	Retinal progenitor cells, stage 1	Mouse	Santa Cruz Biotechnology	sc81311	1/100	[150]
Meis1/2	Retinal progenitors, stages 1, 2	Goat	Santa Cruz Biotechnology	sc10599	1/200	[144]
MITF	RPE	Mouse	Abcam	ab80651	1/500	[182]
MITF	RPE	Mouse	DAKO	M3621	1/200	[150]
MITF	RPE	Mouse	Exalpha Biologicals	X1405M	1/500	[173]
MITF	RPE	Goat	Exalpha Biologicals	X2398M	1/400	[11]
NES	Neuronal and glial surface marker	Mouse	Abcam	ab22035	1/200	[71]
NES	Retinal progenitors, stage 1	Goat	Sigma-Aldrich	N5413	1/200	[11]
NeuN	Retinal progenitors, stage 1	Mouse	Millipore	Mab377	1/500	[101]
NR2E3	Rod progenitors, stage 2	Mouse	Abcam	ab172542	1/300	[10]
NRL	Rod progenitors, stages 1,2	Goat	R&D Systems	af2945	1/200-1/300	[10, 11, 101]
NRL	Rod progenitors, stage 1,2	Goat	Santa Cruz Biotechnology	sc10971	1/50	[11]
Opsin	Opsin, stage 3	Mouse	Sigma-Aldrich	O4886	1/400	[31]
OPN1SW	Opsin blue, stage 3	Rabbit	Millipore	ab5407	1/200-1/500	[10, 31, 50, 101, 150]
OPN1SW	Opsin blue, stage 3	Goat	Santa Cruz Biotechnology	sc14363	1/150-1/200	[11, 34, 72, 173, 182]
OPN1LW/MW	Opsin red/green, stage 3	Rabbit	Millipore	AB5405	1/200-1/500	[10, 11, 31, 32, 34, 50, 101, 182]
OTX2	Photoreceptor, bipolar, and horizontal cell progenitors, stage 1	Goat	R&D Systems	af1979	1/20-1/500	[101], [144]
OTX2	Photoreceptor, bipolar, and horizontal cell progenitors, stage 1	Rabbit	Millipore	ab9566	1/2000	[150]
PAX6	Photoreceptor, glial, amacrine and horizontal cells progenitors, stage 1	Rabbit	Biolegend	901,301	1/300	[144]
PAX6	Photoreceptor, glial, amacrine and horizontal cells progenitors, stage 1	Rabbit	Millipore	ab2237	1/1000	[50, 150]
PAX6	Photoreceptor, glial, amacrine and horizontal cells progenitors, stage 1	Rabbit	Covance	PRB278	1/00-1/300	[31]
PAX6	Photoreceptor, glial, amacrine and horizontal cells progenitors, stage 1	Mouse	Santa Cruz Biotechnology	sc53108	1/200	[116]
PCTN	Basal bodies, staged 2,3	Rabbit	Abcam	ab4448	1/500	[182]
PDE6a	IRD-causative gene	Rabbit	Abcam	ab5659	1/1000	[101]
PCN	Cilia, inner segment, stages 2,3	Mouse	Abcam	ab28144	1/500	[10]
PKCα	Bipolar cells, stages 2,3	Mouse	BD Biosciences	610,107/8	1/100-1/200	[11, 32]
PKCα	Bipolar cells, stages 2,3	Rabbit	Abcam	ab32376	1/200	[11, 71]
PKCα	Bipolar cells, stages 2,3	Rabbit	Millipore	P4334	1/500-1/1000	[182]
PKCα	Bipolar cells, stages 2,3	Rabbit	Sigma-Aldrich	P4334	1/500-1/2000	[10, 34, 50]
PKCα	Bipolar cells, stages 2,3	Rabbit	Santa Cruz Biotechnology	sc17769	1/2000	[150]
PKCα	Bipolar cells, stages 2,3	Rabbit	Santa Cruz Biotechnology	sc208	1/500	[72, 173]
PKCα	Bipolar cells, stages 2,3	Goat	Santa Cruz Biotechnology	sc208G	1/500	[11]
PMEL17	RPE	Mouse	Novus Biologicals	NBP244520	1/500	[182]
PROX1	Horizonal cells, stages 2,3	Rabbit	Millipore	ab5475	1/1500	[32]
PROX1	Horizonal cells, stages 2,3	Rabbit	Millipore	abN278	Jan-00	[72]
PSD95	Postsynaptic adaptor, photoreceptor, stage 3	Mouse	Millipore	Mab1598	1/200	[31]
PVALB	Postsynaptic adaptor, photoreceptor, stage 3	Rabbit	Sigma-Aldrich	P3088	1/200	[11]
RAX	Photoreceptor progenitors, stages 1,2	Mouse	Santa Cruz Biotechnology	sc271889	1/200	[116]
RAX	Photoreceptor progenitors, stages 1,2	Rabbit	Aviva Systems Biology	ARP31926	1/200	[31]
RAX	Photoreceptor progenitors, stages 1,2	Mouse	Sigma-Aldrich	Sab1405061	1/100	[31]
RB	Retinoblastoma-causative gene	Mouse	BD Biosciences	554,136	1/400	[144]
RB	Retinoblastoma-causative gene	Rabbit	Abcam	ab39689	1/500	[144]
RCVN	Photoreceptor progenitors, stage 2 Photoreceptors, stage 3	Rabbit	Millipore	ab5585	1/200-1/2000	[10, 31, 32, 34, 71, 101, 150, 160, 182], [144]
RHO	Photoreceptors, stage 3	Mouse	Millipore	ab5356	1/1000	[101]
RHO	Photoreceptors, stage 3	Rabbit	Millipore	ab9279	1/250	[150]
RHO	Photoreceptors, stage 3	Mouse	Millipore	MabN15	1/100-1/500	[10, 31, 50, 71]
RHO	Photoreceptors, stage 3	Mouse	Sigma-Aldrich	R5403	1/1000	[11]
RHO	Photoreceptors, stage 3	Mouse	Santa Cruz Biotechnology	sc57432	1/200	[31, 32, 72, 173]
RIBEYE	Ribbon synapse, stage 3	Rabbit	Synaptic Systems	192,103	1/800	[11]
RIBEYE	Ribbon synapse, stage 3	Mouse	BD Bioscience	612,044	1/100-1/500	[32, 50]
ROM1	OS, stage 3	Rabbit	ProteinTech	219841AP	1/200	[173]
RP2	IRD-causative gene and plasma membrane/basal body	Rabbit	ProteinTech	141511AP	1/200	[71]
RPE65	Pigment epithelial cells	Mouse	Abcam	ab78036	1/100	[173]
SOX9	Photoreceptor progenitors, stage 1	Mouse	Millipore	ab5535	1/1000	[150]
Synaptophysin	Photoreceptor progenitors, stage 1	Mouse	Sigma-Aldrich	S5768	1/100	[31]
Syntaxin	Outer plexiform layer marker	Mouse	Sigma-Aldrich	S0664	1/200	[31]
TOM20	Mitochondria	Mouse	Santa Cruz Biotechnology	sc17764	1/150	[71]
TOMM20	Mitochondria	Mouse	Abcam	ab56783	1/400	[11]
TRPM1	ON bipolar cells	Rabbit	Atlas Antibodies	HPA014779	1/200	[11]
TUBB3	Cytoskeletal marker, RGC, stage 2	Mouse	Covance	MMS435P	1/800	[31]
TUBB3	Cytoskeletal marker, RGC, stage 2	Mouse	Neuromics	MO15013	1/500	[101]
VGLUT	Postsynaptic marker, photoreceptor terminals, stage 3	Guinea Pig	Millipore	ab5905	1/2000	[10]
Vimentin	Müller glia, stages 2,3	Rabbit	Abcam	ab92547	1/400	[32]
VSX2	Retinal progenitors or bipolar cells, stages 1, 2	Rabbit	Sigma-Aldrich	a96476	1/200	[144]
VSX2	Retinal progenitors or bipolar cells, stages 1, 2	Sheep	Abcam	ab16142	1/200-1/500	[34, 182]
VSX2	Retinal progenitors or bipolar cells, stages 1, 2	Rabbit	Sigma-Aldrich	HPA003436	1/50–1/200	[31, 32]
VSX2	Retinal progenitors or bipolar cells, stages 1, 2	Sheep	Exalpha Biologicals	X1179P	1/200	[10]
VSX2	Retinal progenitors or bipolar cells, stages 1, 2	Mouse	Santa Cruz Biotechnology	sc365519	1/2000	[150]
ZO1	RPE	Rabbit	Abcam	ab59720	1/400	[11]
ZO1	RPE	Rabbit	Thermo Fisher Scientific	c617300	1/100	[173]
ZO1	RPE	Goat	Invitrogen	PA519090	1/250	[150]

The table includes a list of antigens, alongside with indication of the target structure or applicability, host species, where it can be purchased, which dilutions it was used at, and reference to the papers the antibody was used in

IHC can also be adapted for quantitative analysis; for example, the survival of cells in ONL in retinal organoids can be measured, either as cell counts or ONL thickness. This method was used to show photoreceptor degeneration in the ONL of retinal organoids derived from a patient with a mutation in *RP2* and CRISPR-mediated *RP2* knock-out. The cell death also coincided with an increase in apoptotic cells visualised by a TUNEL assay where the 3′ ends of the fragmented DNA are labelled with fluorescently tagged dUTP nucleotides. Significantly more TUNEL-positive cells were present in the ONL at D150 in the patient and knock-out organoids compared to the control which pinpointed a peak in photoreceptor cell death, with subsequent thinning of the ONL, by D180 [71].

While severe photoreceptor layer abnormalities or large cysts are visible under bright field microscopy [103], this technique cannot resolve deep into the tissue. Similarly, the IHC method requires sectioning of the organoid, which proves challenging when the intricate 3D structure and interplay between cell layers are of interest. Multi-photon microscopy improves the image quality deeper into the tissue without the need for sectioning. It confines the emitted fluorescence to a small area by combining the multiple low-energy excitation photons precisely in time and space [145]. The use of multi-photon microscopy allows for live imaging of the invagination process in the forming retinal organoid [23], revealed an interkinetic nuclear migration of retinal progenitors in the D20 optic-vesicle epithelium [24], and showed the differences in M/L-opsin and the rhodopsin marker distribution on the surface of mature organoids [10]. Another method of in-depth imaging, light-sheet fluorescence microscopy, illuminates the sample perpendicular to the objective lens with a thin sheet of excitation light, ensuring that the emitted signal arises only from the in-focus regions [146]. Light-sheet fluorescence microscopy helped visualise the ribbon synapse network formed by photoreceptors and bipolar cells alongside the morphology of photoreceptor basal body and inner segments [147]. A major limitation of the multiphoton imaging of live organoids over longer periods of time is the photodamage to the tissue caused by the excitation light and the need for intrinsic fluorescent markers, potentially necessitating the production of reporter cell lines.

A technique called optical coherence tomography (OCT) allows non-invasive imaging in the eye in vivo and can also be applied to organoids to reveal the internal organisation via the difference in light wave reflectance between nuclear and plexiform layers. An appearance of a layer with high reflectance was reported at D150 near the outer laminated edge of the organoid [127] while another group observed the appearance of alternating high and low reflectance layers at approximately the same stage [10].

The depth of imaging with the multiphoton technique is limited by the penetration of the excitation. Clearing is a chemical treatment that equalises the reflection indexes of the cell membranes and cytoplasm, thus minimizing light scattering, and allows light to travel through a thicker tissue while preserving its excitation and emission efficiency. PACT (passive clarity technique) clearing requires organoids to be fixed with paraformaldehyde, and embedded into hydrogel monomer, after which the resulted hydrogel-organoid hybrid is cleared from the membrane’s lipids [147, 148]. Alternatively, during the 3DISCO (3D imaging of solvent-cleared organs) procedure, the tissue is dehydrated with increasing concentrations of tetrahydrofuran and then impregnated with optical clearing agent dibenzyl ether [149]. The former technique enabled imaging of whole mature organoids at D195 with recoverin, rhodopsin, and cone arrestin staining [150]. During the clearing procedures, the tissue is dehydrated or impregnated with a chemical agent. This can cause a heterogeneous tissue expansion or shrinkage, which might disturb tissue organization. However, a homogeneous tissue expansion of the organoid has the potential to provide a higher resolution imaging of some organelles through expansion microscopy. Second, the stringent chemical treatment might disturb specific antigens on the cleared tissue, limiting the number of useful antibodies. Furthermore, intact organoid clearing requires one specimen per stain which is demanding on production and replicates.

Some potential future applications, such as high throughput testing of drugs or gene therapies, demand an increase in the analysis speed and screening capacity. With an estimated capacity of 200,000 samples per day, a 3D automated reporter quantification (3D-ARQ) platform combines a microplate reader and an excitation/emission detection for the quantitative screening of retinal organoids stained with fluorescent dyes, for example Calcein AM dye that accumulates in the cytoplasm of live cells and is used in viability assays, or JC-1 that allows visualization of the mitochondria rich inner segments of photoreceptors [151].

Often, the subcellular structures of the organoids are of interest, for example, the organization of the membranes inside the OS of photoreceptors, or the structure of the cilium. Light microscopy cannot resolve subcellular structures due to the limited resolving power, whereas transmission electron microscopy (TEM) utilises electrons instead of light and can achieve resolution well below 1 nm. However, it requires elaborate sample preparation with the organoid section undergoing fixation, dehydration, and resin embedding. Imaging with TEM revealed photoreceptor-characteristic organisation and structures, such as the mitochondria rich inner segment, connecting cilium, basal bodies, adherens junctions at the OLM, and microvilli along with membranous disks inside immature OS [10, 17, 26, 71, 78, 129, 152]. TEM imaging also showed the emergence of a ribbon synapse between photoreceptors and bipolar cells in the inner plexiform layer which suggests the functional maturation of retinal organoids [10, 16, 26, 153]. Correlative light and electron microscopy (CLEM) can be used to study the same regions of cells by both light microscopy and TEM and has been used to study retinal organoids to provide ultrastructural information, as well as antigen localisation by immuno-EM [154]. Overall, microscopy is a widely used technique for retinal organoid analysis as it provides an insight into their morphological, molecular, and, to some extent, functional properties.

### Functional analysis

Light responsiveness and synaptic transmission are two crucial features of the functional maturation of photoreceptors and the retina. The light response of a cell can be accessed by measuring a change in its ionic currents in response to light using a patch-clamp. With the patch-clamp, the glass micropipette filled with an electrolytic solution serves as a recording electrode. The electrode touches the membrane of the cell and the suction is applied to establish a tight contact between the pipette and the membrane. This forces the flow of ionic current across the sealed patch of the cell membrane to enter the pipette [155, 156]. Photoreceptor cells may also be stimulated by chemicals, such as cGMP, a secondary messenger in the phototransduction cascade, that potentially mimics the dark current, non-excited state of photoreceptors [157]. In early development, the neurotransmitter GABA is depolarizing and later in development becomes inhibitory; thus the response to it indicates emerging of a functional neuronal network [158]. The patch-clamp recording of recoverin-positive cells in D100 organoids, recorded elevated resting potential and current when compared to control cells, and also the outward current was suppressed with tetraethylammonium, signifying a current that is consistent with photoreceptor electrophysiology [159]. Another study showed that in single and pooled organoids at D150, the response to light, cGMP, and GABA stimulation of RGCs was similar to the developing retina. These experiments require the addition of 9 or 11-*cis* retinal, a chromophore essential for rhodopsin activity, which also serves as a control for the response being opsin-mediated [17]. The OS containing cells of retinal organoids were reported to be comparable to rods of mice retina before eye-opening in their membrane capacity, potential, and resistance recordings. A whole-cell recording of presumed photoreceptors seemed to exhibit detectable hyperpolarization-activated current and the current/voltage curve resembles that of rods [102]. Although the patch-clamp technique is limited by low experimental throughput, it has an important advantage of recording a photoreceptor-specific signal.

Another method to investigate the functionality of retinal organoids utlises confocal or multi-photon microscopy to measure changes in intracellular Ca^2+^ indicative of the inward dark current that is characteristic of photoreceptors. The fluorescent calcium dye Fura-2 allows for visualization of the increase Ca^2+^ influx in response to cGMP-analogue in cells of D45 and D90 [31], D175 [150], and in cell surface antigen CD73-positive photoreceptors of D190 retinal organoids [50]. In cells of retinal organoids that were genetically modified to express the fluorescent calcium sensor GCaMP6s, a change in Ca^2+^ influx in response to light stimulation was recorded in photoreceptors and in OFF bipolar cells but very few ON bipolar cells [11].

Two-photon microscopy has been combined with patch-clamp electrophysiological recordings to test membrane localization and functionality of optogenetic constructs in retinal organoids. Several types of light-sensitive microbial opsins were expressed in cells of the retinal organoids and excitatory photocurrents were observed for the depolarizing opsins alongside inhibitory photocurrents for the hyperpolarizing opsins [160].

Micro-electrode arrays (MEAs) contain multiple electrodes embedded into a ceramic or glass carrier [161]. Retinal organoids are placed with RGCs facing down onto the MEA and a light or chemical stimulus is applied, resulting in detectable voltage changes [162]. A stronger response to light was observed in control compared to AMD patient-derived organoids [129]. A comparison between the recording of D150 organoids derived control versus a patient with a mutation in *PRPF31,* causing the degeneration of the mid-peripheral retina, implied a significantly reduced response to GABA, but no changes in response to cGMP-analogue [100]. The advantage of the MEA technique is that it measures output from the RGCs which shows inner retinal connectivity; however, the MEA has the challenge of being reliant on the presence of both mature RGCs and photoreceptors at the same stage. This is challenging given the reduction in RGCs by the stage that photoreceptors have developed, as well as the necessity to preserve an inner retinal structure and OS while setting up the MEA. Furthermore, for the MEA, extra care should be taken with the experimental design to eliminate the other sources of electrical signal other than photoreceptors, unlike Ca^2+^ imaging or patch clamping where the origin of the response is more clearly identified.

An important potential addition to testing photoreceptor-driven responses would be to generate a disruption of the elements of the phototransduction pathway, such as by CRISPR/Cas9-mediated knock-out, to confirm the source of the responses. While testing the light responsiveness of retinal organoids in vitro is a “holy grail” of organoid characterisation, there is still a lot of work to be done before it could be used as a routine and reproducible measure of photoreceptor function in disease or following rescue.

### Limitations of retinal organoids as models of the in vivo retina

In the retina, the OS of cone and rod photoreceptors are specialised primary cilia that contain stacks of deep membrane folds of the plasma membrane, or double-membrane disks enclosed by the plasma membrane, respectively [8]. The structure of these disks is important for phototransduction, as they harbour light-sensitive opsins that are conjugated with the chromophore 11-*cis*-retinal. A major challenge in retinal organoid technology to study photoreceptor disease has been to produce properly stacked OS. Mature retinal organoids contain OS-like structures containing membranous disks that are loaded with opsins [154], but lack the correct disk stacking and orientation. Moreover, an extensive characterisation of the observed membranous structures remains to be done [17, 26, 27, 78, 150, 163]. Notwithstanding, retinal organoids can still be instrumental in studying disk and OS biogenesis [19].

OS membrane disks are renewed and shed daily [164, 165]. When shed, they are phagocyted by the RPE cells [166]. The RPE supports the high metabolic rate of the photoreceptors by allowing the flow of nutrients from the choroid [167] and participates in the visual cycle by creating ionic gradients and converting all*-trans*-retinal into 11*-cis*-retinal [168, 169]. During development, the RPE secretes compounds required for photoreceptor maturation [30, 170–172]. Retinal organoids usually contain RPE; however, they are generally juxtaposed to the photoreceptors in a clump, and not in a monolayer like the situation in vivo. Therefore, the challenge remains to generate an optic cup-like structure where RPE and retina co-exist in close proximity. A recently developed retina-on-a-chip microfluidic device reported such a co-culture by the embedding of the retinal organoid in hydrogel within a small distance of RPE for 7 days, but there was only a small region of contact between the three-dimensional organoids and the two-dimensional RPE [173].

Another structure yet to be replicated is the fovea, a substructure in the centre of the human retina that is required for high-acuity colour vision. The absence of rod photoreceptors and other retinal cell types in the foveal pit allows light to directly stimulate densely packed cones. The fovea is specific to human and non-human primates and is selectively affected by diseases such as macular degeneration. The fovea develops postnatally in the area of the retina where the development of rods from late retinal progenitor cells is inhibited, and the location of this area is likely to be determined by transcription factors involved in the definition of the axis of the retina [174]. While the spontaneous induction of the foveal pit might not be likely in the organoid, several studies reported the generation of cone-enriched organoids, possibly by removal of rod-favouring RA [97, 175, 176].

Blood vessel and immune-related cells are also missing from retinal organoids; these are derived from the mesoderm and during development migrate into the developing optic vesicles. The addition of these cells to retinal organoids would likely require assembloid type technology to bring together cells from different developmental lineages, which was pioneered in brain organoids [177]. Recently, a model of outer-blood-retinal barrier on-a-chip has been described combining RPE and endothelial cells in a microfluidic chamber [178].

### Future outlook

The small size and inherent heterogeneity of cell types in retinal organoids pose significant technical challenges for the types of analysis with higher material demand, lower sensitivity, or difficulty to distinguish between the signal coming from different cell types. It is, however, important to realise the potential of the retinal organoids to reveal neuroretina-specific gene regulation, pathological processes, and therapeutic manipulation.

It would be desirable to increase the efficiency and reproducibility of organoid production and decrease the labour required for their production. To do so, first, it is important to understand the reasons for the innate heterogeneity between iPSC lines and why some lines generate organoids more efficiently than others. More reproducible organoid generation from a particular cell line can be achieved by a better understanding of epigenetic control of iPSC pluripotency [179], as well as the neurodevelopmental factors that are involved in neural patterning and cell fate decisions. To improve and accelerate differentiations, organoids may be grown in rotating-wall vessel bioreactors [153, 180]. Also, reduction of the required labour and level of manual skill is desirable. For this, scraping or checkerboard methods that bypass the manual dissection step to help increase the yield of NRVs obtained are potentially useful [181, 182]. One such example, the recently described AMASS (agarose microwell array seeding and scraping), combines a controlled number of cells seeding in a microwell array with a checkerboard scraping. With this method, more than 3000 organoids were obtained in a single well of a 6-well plate [11]. Culture conditions could also mimic physiological conditions better through assembloid methodologies, such as combining cultures of the tissues produced independently to improve cell cross-talk and implement the use of biomaterials for cellular support [177, 183]. Current state-of-the-art differentiation protocols span several months and up to a year, for a fully mature retinal organoid. Organoid or precursor freezing might help address this and facilitate collaborations across laboratories in the field [150]. Ideally, retinal organoids could be shipped at room temperature at any stage for at least 5 days and still maintain their biological activity and functionality [184]. The heterogeneity of the retinal organoid samples should also be considered regarding analyses that are focused on certain cell types. As retinal organoids are small, the cell type of interest might be underrepresented and the useful signal lost due to the noise in individual organoids. It can be enriched by manual dissection [185], by coupling MS with flow cytometry, or via fluorescent reporters [43, 50, 92]. In addition, another way of specific cell-type enrichment would be to minimise the material needed for some procedures by refining their specificity and the signal-to-noise ratio.

In conclusion, we now have a number of state-of-the-art techniques that can facilitate analysis of the retinal organoids at different levels of complexity to understand disease mechanisms and test potential therapies. An expansion of the analytical toolbox will increase the benefit of the retinal organoid model for teasing apart the molecular mechanism of genetic blinding diseases.

## Data Availability

Not applicable.
